# Leaf Segmentation on Dense Plant Point Clouds with Facet Region Growing

**DOI:** 10.3390/s18113625

**Published:** 2018-10-25

**Authors:** Dawei Li, Yan Cao, Xue-song Tang, Siyuan Yan, Xin Cai

**Affiliations:** 1 College of Information Science and Technology, Donghua University, Shanghai 201620, China; daweili@dhu.edu.cn (D.L.); 2171339@mail.dhu.edu.cn (Y.C.); 2171353@mail.dhu.edu.cn (S.Y.); xcai@dhu.edu.cn (X.C.); 2 Engineering Research Center of Digitized Textile & Fashion Technology, Ministry of Education, Donghua University, Shanghai 201620, China

**Keywords:** individual leaf segmentation, point cloud, greenhouse plant, facet over-segmentation, local K-means clustering, region growing

## Abstract

Leaves account for the largest proportion of all organ areas for most kinds of plants, and are comprise the main part of the photosynthetically active material in a plant. Observation of individual leaves can help to recognize their growth status and measure complex phenotypic traits. Current image-based leaf segmentation methods have problems due to highly restricted species and vulnerability toward canopy occlusion. In this work, we propose an individual leaf segmentation approach for dense plant point clouds using facet over-segmentation and facet region growing. The approach can be divided into three steps: (1) point cloud pre-processing, (2) facet over-segmentation, and (3) facet region growing for individual leaf segmentation. The experimental results show that the proposed method is effective and efficient in segmenting individual leaves from 3D point clouds of greenhouse ornamentals such as *Epipremnum aureum*, *Monstera deliciosa*, and *Calathea makoyana*, and the average precision and recall are both above 90%. The results also reveal the wide applicability of the proposed methodology for point clouds scanned from different kinds of 3D imaging systems, such as stereo vision and Kinect v2. Moreover, our method is potentially applicable in a broad range of applications that aim at segmenting regular surfaces and objects from a point cloud.

## 1. Introduction

Leaves account for the largest proportion of all organ areas for most kinds of plants and comprise the main part of the photosynthetically active material in a plant. Leaves contain important information about the surface morphology and structure of a plant; therefore, observation of leaves can reveal its growth status. Changes in leaf morphology, texture, or color normally reflects biotic stress (plant diseases and pests) or abiotic stress (drought), so automatic leaf segmentation, recognition, and classification methods can provide rapid early warnings for agriculture facilities and, ultimately, help to increase plant output. Studies have used traditional imaging techniques and analyzing tools to carry out 2D leaf recognition and classification. Neto et al. used Gustafson-Kessel clustering and a genetic algorithm to segment individual leaves from different environments, and tested the method successfully on soybean, sunflower, pigweed, and velvetleaf plants [1]. Xu et al. extracted various color and texture features of leaves to identify nitrogen- and potassium-deficient tomatoes, and showed that the system could diagnose disease about 6–10 days before experts could reach the same assessment [2]. Kalyoncu and Toygar segmented and classified leaves of different species using a linear discriminant classifier [3], achieving an accuracy of 70% on the Leafsnap dataset [4]. Zhang et al. proposed a novel cucumber disease recognition approach combining three pipelined procedures: segmenting diseased leaf images by K-means clustering, extracting shape and color features from lesion information, and classifying diseased leaf images using sparse representation [5].

Plant phenotyping is intended to measure complex traits related to growth, yield and adaptation to stress with a certain accuracy and precision at different scales of organization, from organs to canopies [6,7]. As an important task in high-throughput phenotyping, individual leaf segmentation has been drawing considerable attention from biology research and agricultural industry facilities. Recently, plant phenotyping software tools have sprouted up, such as PlantCV [8] and Leaf-GP [9]. Automatic leaf segmentation, alignment, and tracking algorithms are the key components of those tools, and are mostly based on 2D imaging. Scharr et al. made a recent survey of state-of-the-art 2D leaf segmentation methods for plant phenotyping, and evaluated the results of four different methods [10]. Given a fluorescence plant video, Yin et al. proposed a method that performed multi-leaf joint segmentation, alignment, and tracking (SAT) for *Arabidopsis* [11]. Viaud et al. proposed an *Arabidopsis* leaf segmentation and tracking system, in which the plant images were first segmented by a watershed algorithm and then refined by an ellipsoid-shaped model [12]. Recently, some researchers have introduced deep learning in counting or segmenting individual leaves from canopy images. Dobrescu et al. used a modified Resnet50 net to count leaves of *Arabidopsis* and tobacco plant images in the Computer Vision Problems in Plant Phenotyping (CVPPP) 2017 Leaf Counting Challenge dataset [13]. Although their method outperforms the winner of the previous CVPPP challenge, it is not able to locate the exact positions of leaves. Morris employed a pyramid-like Convolutional Neural Network (CNN) to segment leaves from images of dense foliage [14], which restricts the segmentation to the leaf boundary searching level.

Current leaf phenotyping algorithms are mostly image-based methods that often leverage machine learning and pattern recognition techniques. Despite the evident progress that has been made, leaf phenotyping remains restricted to processing several kinds of standard crops and plants (e.g., wheat [8,9] and rosette plants [10,12,15]). Moreover, the structure of the canopy is usually complicated, resulting in overlapping among leaves, which poses a great challenge to 2D leaf segmentation algorithms. As the cost of hardware continues to fall and image technology advances, 3D imaging has become a promising solution for sensing and phenotyping in cost-sensitive agricultural applications. Compared to traditional 2D imaging and vision techniques, 3D imaging not only acquires pixel-level colors, but also acquires the most important depth information of the scene. The spatial structure information of the plant is highly beneficial for studying its growth and its phenotypic traits. Recently, research on individual leaf segmentation with depth and range images or 3D point clouds has emerged. Some related literature has focused on tasks such as separating canopy volume from foliage point clouds scanned from a Lidar [16], segmenting single trees from forest point clouds [17,18], and estimating the leaf area density (LAD) of the plant [19]. Teng et al. built a segmentation and classification system for leaves, by which individual leaves can be segmented from point clouds by a joint 2D/3D approach [20]. Chaurasia and Beardsley designed a superpixel graph clustering algorithm to carry out initial segmentation of single-lobed leaves from point clouds, and then used Iterative Closest Point (ICP) matching to refine the segmentation results [21]. Others have focused on low-cost 3D sensors and techniques to generate dense plant point clouds, from which plant organs are further segmented. Li et al. [22] established a low-cost stereo system to reconstruct point clouds from six kinds of greenhouse plants, showing high accuracy and invariance to illumination changes. Paproki et al. [23] used multi-view 3D reconstruction and 3D meshes to generate a model of *Gossypium hirsutum* from 64 images, and then segmented leaves, petioles, and internodes from the model via a pipeline of four steps. Xia et al. exploited the Kinect v1 sensor to carry out in situ leaf segmentation in a greenhouse and reached a total segmentation rate of 87.97% [24]. Several research groups even turned to 4D point cloud data, with time as the 4th dimension for facilitating automatic organ segmentation. Li et al. conducted a spatial and temporal analysis on 4D point cloud data of plants; budding and bifurcation events could be accurately segmented in a non-real-time way [25].

In this paper, we propose an individual leaf segmentation approach for dense plant point clouds using facet over-segmentation and facet region growing. The method can be divided into three steps: (1) point cloud pre-processing; (2) facet over-segmentation; and, (3) facet region growing for individual leaf segmentation. In the first step, the raw point clouds acquired from 3D sensors are filtered and preprocessed to leave out non-leaf areas. Then iterative principle component analysis (IPCA) is used to compute the spatial characteristics of each point in the point cloud (e.g., normal of the fitted plane in a neighborhood, and smoothness). In the second step, we first deploy a number of seed points and then apply a clustering algorithm to the plant point cloud with those seed points being the initial cluster centers to generate small facets. The facets generated have a smooth spatial structure and are uniformly distributed on the point cloud. In the third step, we use facet adjacency and coplanarity as conditions to carry out facet region growing with a breadth-first search strategy. After region growing, multiple facets will be combined to form a larger spatial structure that is finally regarded as a segmented leaf provided that it covers enough points. The experimental results show that the proposed method is effective in segmenting individual leaves from greenhouse ornamentals such as *Epipremnum aureum*, *Monstera deliciosa*, and *Calathea makoyana*, with precision and recall above 90%. The results also reveal the wide applicability of the proposed methodology for point clouds scanned from different kinds of 3D imaging systems, such as stereo vision and Kinect v2.

## 2. Materials and Methods

### 2.1. Platform and Experiment Subjects

#### 2.1.1. Platform

**Figure 1:**
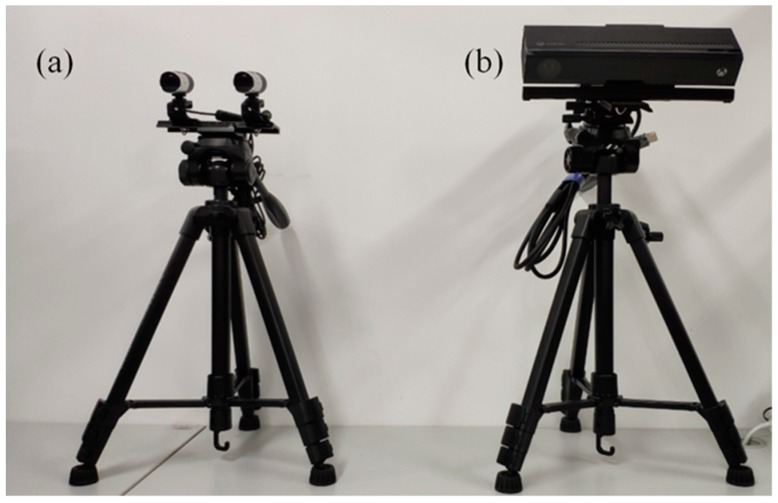
Two types of imaging sensors with tripods: (**a**) shows the binocular stereo vision system used in this research; (**b**) shows a Kinect V2 sensor mounted on a tripod.

The processing unit was a desktop, assembled by the authors, with an Intel Core i7-7700 CPU (Intel, Santa Clara, CA, USA) and 16 GB RAM. The software environment included VS2013 (Microsoft, Redmond, WA, USA) with the Point Cloud Library (PCL) [26], which were all operated in Windows 10. In the experiment, two types of imaging sensors with tripods were adopted to scan sample plants for point clouds. The first system was a binocular stereo vision system proposed in [22] (as illustrated in Figure 1a). This stereo vision platform consisted of two high-definition webcams (HD-3000 series, Microsoft, Redmond, WA, USA), a supporting board (LP-01, Fotomate, Jiangmen City, China) with a scale line, and a tripod (VCT-668RM, Yunteng Photographic Equipment Factory, Zhongshang City, China). The second sensor was a structured light sensor [27] (Kinect V2, Microsoft, Redmond, WA, USA) that obtains depth information by capturing reflections from the projected infrared light pattern. The Kinect sensor was mounted on the same type of tripod as used for the stereo system (as illustrated in Figure 1b).

#### 2.1.2. Experiment Subjects

Three types of greenhouse ornamentals were adopted as research subjects in this paper: *Epipremnum aureum* (Linden & André) G.S. Bunting, *Monstera deliciosa* L., and *Calathea makoyana.* The Kinect V2 sensor was used to acquire the point cloud of an *Epipremnum aureum* sample plant and a *Calathea makoyana* sample plant. The binocular stereo vision system was used to reconstruct the 3D point cloud of a *Monstera deliciosa* sample plant.

### 2.2. Framework

**Figure 2:**
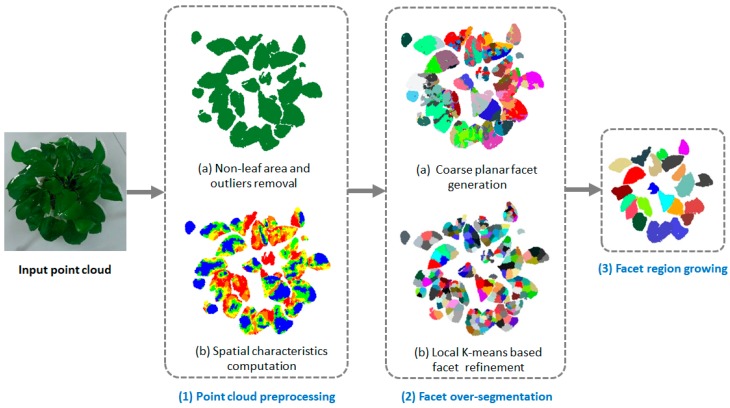
Overview of the proposed method.

Our individual leaf segmentation approach for plant point clouds consisted of three steps; the overview is shown in Figure 2. The first step was the pre-processing of the captured point cloud. Since the point cloud acquired from a 3D sensor has a large amount of noise points and non-leaf areas, such as pots and the ground, it was necessary to filter the original plant point cloud to generate a point cloud containing only leaves for further processing. In addition, the spatial characteristics of each point were also calculated in the pre-processing step, including the normal of the fitted plane in the neighborhood and the smoothness. In the second step, we carried out facet over-segmentation on the preprocessed point cloud data. In this step, a large number of uniformly distributed 3D facets that have a flat spatial structure were generated on the point cloud. The aim of over-segmentation is to cluster the points that share the same local spatial characteristics in advance. Therefore, it is a fine solution for the issue of individual leaf segmentation because aggregating bigger structures, such as facets to a leaf, is much easier than directly aggregating original points to a leaf in the cloud. We first utilized the computed spatial characteristics of each point in the first step to coarsely cluster the leaf points that are nearby and coplanar into the same facet. Then, to make the boundaries of over-segmented facets more regular, we employed local K-means clustering to refine all facets. In the third step, we realized individual leaf segmentation based on a facet region growing strategy. In this step, adjacency and co-planarity among facets were the conditions of facet region growing. After region growing, multiple facets were combined to form a larger spatial structure that could finally be regarded as a segmented leaf if it covers a sufficient number of points.

### 2.3. Point Cloud Pre-Processing

#### 2.3.1. Removal of Non-Leaf Areas and Outliers

During the 3D point cloud reconstruction process, most imaging sensors will perform interpolation in certain local areas of the original point cloud data, causing the boundary points of different planes in the point cloud to leap across different surfaces. Although interpolation smoothed the measurement errors for interior points, the spatial variances of boundary points became much larger than those of the interior points, which may result in connection of leaves that are actually isolated. Therefore, the removal of outliers in the point cloud is an important step in the framework. The paper applied different filtering methods on point clouds generated by two types of imaging methods. Three filtering operations were employed on the point cloud of the *Epipremnum aureum* sample plant. Firstly, we filtered out the points belonging to the ground by using the z-axis coordinates of the space. Second, if the number of points in the sphere of radius r centered at the current point was lower than a threshold $n_{1}$, then the current point will be considered an outlier and discarded (i.e., the RadiusOutlierRemoval function in the PCL library). Lastly, we calculated the average distance between the k nearest neighboring points and the current point, and removed the neighboring point whose distance was larger than an upper bound derived by adding the average spacing to a coefficient $n_{2}$ multiplied by the standard deviation (the StatisticalOutlierRemoval function in the PCL library). Consequently, non-leaf points and outliers in the *Epipremnum aureum* point cloud were filtered. Only the first and the third operations were performed on the *Monstera deliciosa* point cloud to carry out filtering. Only the first and the second filtering operations were performed on the *Calathea makoyana* point cloud; there was no need to apply the last filtering operation because the leaves of *Calathea makoyana* are more regular than the leaves of *Epipremnum aureum*.

#### 2.3.2. Using IPCA to Compute Spatial Characteristics of Each Point

We specify the spatial characteristics of each point $x_{i}$ in the point cloud χ in two parts: the normal $n_{i}$ of the fitted plane in a neighborhood of $x_{i}=[x_{i},y_{i},z_{i}{]}^{T}$ and smoothness $s_{i}$ of $x_{i}$. The fitted plane $f_{i}=(X_{3\times K},x_{i},n_{i})$ of $x_{i}$ is a 3-tuple, in which the K nearest neighborhood matrix $X_{3\times K}$ (including $x_{i}$ itself) of $x_{i}$ is calculated by iterative principle components analysis (IPCA). In each iteration, a plane passing through $x_{i}$ will be re-estimated by performing PCA on the inliers in the neighborhood. The notation d(i,j) is defined as the Euclidean distance from the point $x_{j}$ in $X_{3\times K}$ to the fitted plane $f_{i}$ of $x_{i}$. If d(i,j) is larger than the fixed threshold parameter $\sigma_{1}$, point $x_{j}$ will be removed from $X_{3\times K}$ before next iteration. The calculation of d(i,j) is as follows:

(1) $$d\left(i,j\right)=\frac{\left|n_{i}{}^{T}\cdot (x_{j}-x_{i})\right|}{\left|n_{i}\right|}\;$$

The IPCA iterative process will cease when the size of $X_{3\times K^{\prime }}$ remains unchanged, which also means $K^{\prime }$ decreases from K and finally becomes stable. The fitted plane $f_{i}=(X_{3\times K^{\prime }},x_{i},n_{i})$ calculated by IPCA will be very helpful in representing the spatial structure around each point $x_{i}$. In IPCA, the three-by-three covariance matrix $C_{i}$ of $X_{3\times K}$ needs to be calculated for updating the fitted plane:

(2) $$C_{i}=\frac{1}{K}X_{3\times K}\cdot X_{3\times K}^{T}\;$$

In Equation (2), the data matrix $X_{3\times K}$ has been centralized. $\lambda_{1}$, $\lambda_{2}$, and $\lambda_{3}$ are the three descending eigenvalues of the covariance matrix $C_{i}$ formed by $X_{3\times K}$. Therefore, the unit eigenvector corresponding to $\lambda_{3}$ can be regarded as the normal $n_{i}$ of the fitted plane of $x_{i}$. The smoothness indicator $s_{i}$ is defined as the ratio between $\lambda_{2}$ and $\lambda_{3}$ [28,29]. The greater the smoothness, the flatter the neighborhood of $x_{i}$. Both the normal $n_{i}$ and the smoothness $s_{i}$ will be updated in each iteration of IPCA. The pseudocode of the IPCA algorithm is given in Algorithm 1.

**Algorithm 1** IPCA for computing the spatial characteristics of each point.**Input:** Point Cloud χ, $x_{i}$ is any point in χ.**Parameters**: Initial number of points in the neighborhood K.**Output:** The unit normal vector $n_{i}$, and the smoothness $s_{i}$.1**for** each point $x_{i}$ in χ
**do**2 Initialize $x_{i}$’s K-nearest neighbors data matrix $X_{3\times K}$.3 **repeat**4  Compute the covariance matrix $C_{i}$ of $X_{3\times K}$ by Equation (2).5  Compute the eigenvalues in descending order $\lambda_{1}$, $\lambda_{2}$, and $\lambda_{3}$, and their corresponding eigenvectors $v_{1}$, $v_{2}$, and $v_{3}$ of $C_{i}$ by Eigenvalue Decomposition.6   $n_{i}\leftarrow v_{3}$, $s_{i}\leftarrow \lambda_{2}/\lambda_{3}$.7  Compute the distance d(i,j) between the point $x_{j}$ and $x_{i}$’s current fitted plane by Equation (1).8   **if**
$d(i,j)>\sigma_{1}$
**then**9    remove the point $x_{j}$ from $X_{3\times K}$10   **end if**11  **until**
$X_{3\times K}$ remains the same12
**end for**


### 2.4. Facet Over-Segmentation

#### 2.4.1. Seed Point Selection and Coarse Planar Facet Generation

The normal $n_{i}$ of the fitted plane in the neighborhood and the smoothness $s_{i}$ of $x_{i}$ in the point cloud χ now have been computed according to Section 2.3.2. The coarse facet generation will be performed based on the normal and smoothness indicators. The essence of the process is to cluster the points in the point cloud with the same spatial characteristics. We carry out this coarse clustering by deploying a number of seed points first and then clustering similar points in a local neighborhood to each seed point. In order to produce reasonable seed points, we randomly select one point $x_{i}$ in χ and let the point $x_{j}$ with the greatest smoothness in the K-nearest neighborhood $X_{3\times K}$ of $x_{i}$ to be a seed point. Next, based on the seed point $x_{j}$, a facet whose points have the same spatial characteristics will be established by local region growing. There are three conditions for local point region growing: (i) the Euclidean distance between a candidate grow point $x_{k}$ and the seed point $x_{j}$ must be less than a threshold $r_{1}$; (ii) the angle between the normals of the fitted planes of the seed point $x_{j}$ and $x_{k}$ needs to be less than θ; and (iii) the distance from the point $x_{k}$ to the fitted plane $f_{j}$ of $x_{j}$ should be less than $\sigma_{1}$. The above conditions are used to judge whether $x_{k}$ and $x_{j}$ are near and coplanar, and the three conditions must be satisfied at the same time to allow the facet region of $x_{j}$ to include $x_{k}$. If a point $x_{k}$ has been included in a facet by the local region growing, then it will be labelled as used and extracted from χ. With the help of the above procedure, coarse planar over-segmented facets will be generated. The detailed pseudocode for this step is given in the first half of Algorithm 2.

#### 2.4.2. Local K-Means Clustering Based Facet Refinement

In Section 2.4.1, points in χ with the same spatial characteristics have been coarsely clustered into over-segmented facets, and we have also obtained the collection $\chi_{\mathrm{seed}}$ of seed points. The size of facets can be controlled by the parameters K and $r_{1}$; the parameter tuning process will be further discussed in Section 3. The idea of our local K-means clustering is similar to simple linear iterative clustering (SLIC) [28].

For facets refinement, a seed point $x_{\mathrm{seed}}$ will be first randomly selected from $\chi_{\mathrm{seed}}$ and then a search sphere of radius $r_{2}$ centered at $x_{\mathrm{seed}}$ is formed. It should be noted that the sphere may possibly contain multiple seed points in $\chi_{\mathrm{seed}}$. In each sphere, the Euclidean distances from each point $x_{i}$ in χ to all seed points (if there are any) will be calculated respectively. After all seed points in $\chi_{\mathrm{seed}}$ have been processed with the above measure, each point $x_{i}$ in χ is classified to its nearest seed point. After all points in the cloud have been classified, each cluster now becomes a new facet, and the position of each seed point will be updated as the mean of the points in the facet it belongs to. We repeat the above local K-means clustering until the cluster centers of facets become stable. In experiments, we have observed that the boundaries of facets became stable after 3 to 5 iterations on several kinds of point clouds. Therefore, we fix the maximum number of iterations for local K-means to be 10. The detailed pseudocode for facet refinement is given in the second half of Algorithm 2.

**Algorithm 2** Facet over-segmentation.**Input:** Unit normal vector $n_{i}$, and the smoothness $s_{i}$ of each point $x_{i}$.**Parameters**: $r_{1}$, θ, and $\sigma_{1}$**Output:** the collection F of facets, the seed point set $\chi_{\mathrm{seed}}$.1
$\chi_{\mathrm{seed}}\leftarrow \emptyset$
2**for** each unused point $x_{i}$ in χ
**do**3 set the point $x_{j}$ with the largest $s_{j}$ in $X_{3\times K}$ of $x_{i}$ as the seed point $x_{\mathrm{seed}}$4 $\chi_{\mathrm{seed}}.push\_back(x_{\mathrm{seed}})$5 **for** each unused point $x_{k}$ in χ
**do**6  **if**
$x_{k}$ and $x_{\mathrm{seed}}$ satisfy the three conditions at the same time.7   (i) $\left|x_{k}-x_{\mathrm{seed}}\right|\leq r_{1}$8   (ii) $\mathrm{acos}(n_{k}\cdot n_{\mathrm{seed}})\leq \theta$9   (iii) $d(seed,k)\leq \sigma_{1}$10   **then grow**
$x_{k}$ belongs to the region of $x_{\mathrm{seed}}$, and label $x_{k}$ as used11    **end if**12   **end for**13   **end for**14   Set each region of $x_{\mathrm{seed}}$ in $\chi_{\mathrm{seed}}$ as a facet.15   Set distance $d_{i}=\infty$ for each point $x_{i}$ in χ.16   **repeat**17      **for** each cluster center $x_{\mathrm{seed}}$ in $\chi_{\mathrm{seed}}$
**do**18       **for** each point $x_{i}$ in a sphere of radius $r_{2}$ centered at $x_{\mathrm{seed}}$
**do**19       Compute the distance $D=\left|x_{\mathrm{seed}}-x_{i}\right|$ (multiple seeds may exist)20        **if**
$D\leq d_{i}$
**then**21         $d_{i}\leftarrow D$22         classify $x_{i}$ to the cluster of $x_{\mathrm{seed}}$23        **end if**24       **end for**25      **end for**26     Points that do not belong to any facet are classified to its nearest seed points. Each cluster of a seed $x_{\mathrm{seed}}$ now becomes a new facet $f_{\mathrm{seed}}$, and all facets form a collection F.27    **until** the positions of seeds remain stable.

### 2.5. Facet Region Growing for Individual Leaf Segmentation

In Section 2.4, we have accomplished facet over-segmentation and obtained the collection F of refined facets. The main idea of this section is to carry out facet region growing from each facet $f_{i}$ in F according to the facet adjacency and the coplanarity. Then, multiple facets will be combined into a larger structure that can be finally regarded a segmented individual leaf if it covers a sufficient number of points.

**Figure 3:**
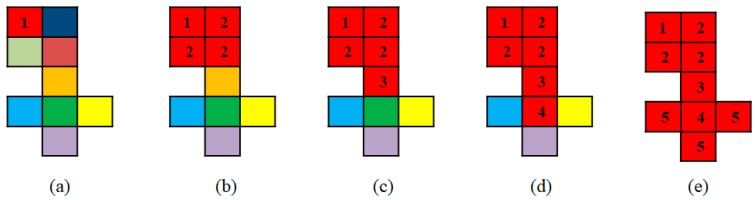
Breadth-first searching strategy for facet region growing: (**a**) shows the facets before region growing started; (**b**) shows the result of the first round of facet region growing; (**c**) shows the result of the second round of facet region growing; (**d**) shows the result of the third round of facet region growing; (**e**) shows the result of the final round of facet region growing.

The facet region growing from a starting facet $f_{i}$ to a facet $f_{j}$ must satisfy two conditions: (i) the facet $f_{j}$ is adjacent to $f_{i}$; and. (ii) if $x_{i}$ and $x_{j}$ are the centers of the two facets respectively, d(i,j), which represents the distance from $x_{j}$ to the facet $f_{i}$, is less than a threshold $\sigma_{2}$. If facets $f_{j}$ and $f_{i}$ satisfy both of these conditions, they are regarded as being located on the same leaf. A breadth-first search strategy is employed in the facet region growing, which is demonstrated by Figure 3. In the breadth-first facet region growing, we begin by defining a starting facet labeled by the number “1” in Figure 3a. The first round of facet growing incorporates three nearby facets with label “2” into the region (Figure 3b). Finally, after 4 rounds of growing, the total structure now covers 9 small facets that are assigned the same red color (Figure 3e). The pseudocode for facet region growing is listed in Algorithm 3.

**Algorithm 3** Facet region growing.**Input:** The collection of facts F.Parameters: $\sigma_{2}$**Output:** The collection of individual leaves.1**for** each unused facet $f_{k}$ in F
**do**2 a temporal facet queue for breadth-first search A←NULL3 Set $f_{k}$ as the starting facet of a new individual leaf.4 $A.push\_back(f_{k})$, and label $f_{k}$ as used.5 **repeat**6  $f_{i}=A.pop\_front$7  **for** each unused facet $f_{j}$ in F
**do**8    **if**
$f_{j}$
**is adjacent and**
$d(i,j)\leq \sigma_{2}$
**then**9     $A.push\_back(f_{j})$ and label $f_{j}$ as used10    Grow $f_{i}$ to $f_{j}$.11   **end if**12  **end for**13 **until**
A=NULL14
**end for**


## 3. Results and Discussion

### 3.1. Point Cloud Pre-Processing Results

**Figure 4:**
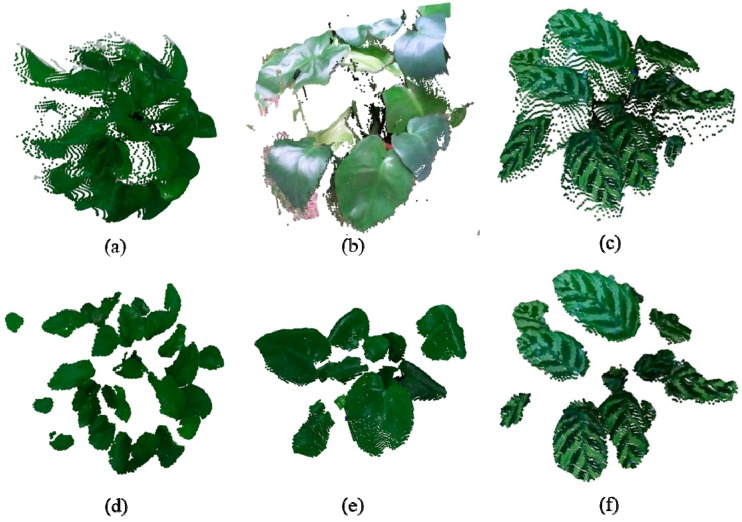
The original and pre-processed point clouds of the three greenhouse sample plants: (**a**) the original point cloud of *Epipremnum aureum*; (**b**) the original point cloud of *Monstera deliciosa*; (**c**) the original point cloud of *Calathea makoyana*; (**d**) the pre-processed result on *Epipremnum aureum*; (**e**) the pre-processed point cloud on (**b**); and, (**f**) the preprocessed result on (**c**).

The Kinect V2 sensor is used to acquire point clouds of an *Epipremnum aureum* sample plant and a *Calathea makoyana* sample plant, each containing a total of 217,088 points. The binocular stereo vision system is used to reconstruct the 3D point cloud of a *Monstera deliciosa* sample plant that contains 200,000 points. Three filtering operations are employed on the point cloud of the *Epipremnum aureum* sample plant. Firstly, we filter out the non-leaf points according to greenness and the z-axis coordinates of the point cloud. Secondly, we compare the number of points in the radius r=0.015m around each point with a threshold $n_{1}=85$. If the number of points is lower than 85, the current point will be considered an outlier and discarded. Last, we calculate the average distance between the k=40 nearest neighboring points and the current point, removing the neighboring points whose distance is larger than an upper bound of adding the average spacing to $n_{2}=1$ multiplied by the standard deviation. However, only two filtering operations are performed on the *Monstera deliciosa* point cloud, with parameters set as k=25 and $n_{2}=1$. Generally speaking, the radius-based filtering method can better preserve the dense parts of the point cloud, while the filtering method based on the average distance within a neighborhood is well suited for removing isolated outliers. The original and the pre-processed point clouds are shown in Figure 4.

### 3.2. Results of Facet over-Segmentation

**Figure 5:**
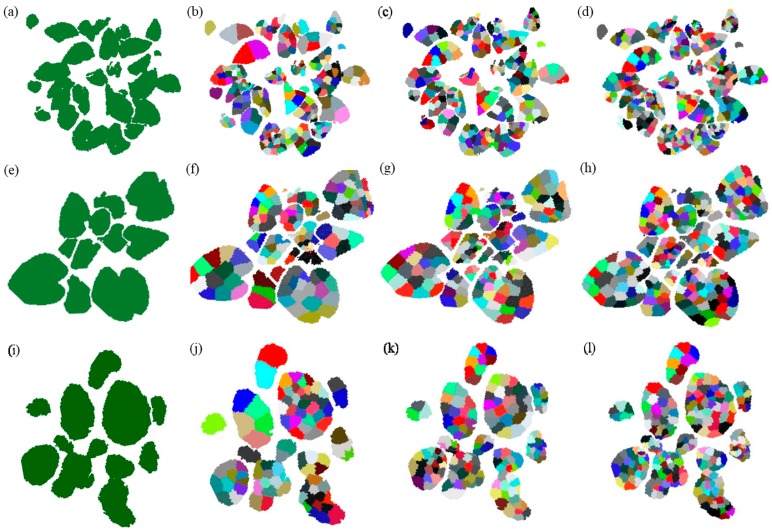
The facet over-segmentation of *Epipremnum aureum, Monstera deliciosa, and Calathea makoyana*; point clouds (**a**,**e**,**i**) show the top-views of the original three point clouds, respectively; (**b**) the over-segmentation result of *Epipremnum aureum* when K is 100 and $r_{1}$ is 0.05 m; (**c**) the result when K is 40 and $r_{1}$ is 0.05 m; (**d**) the result when K is 20 and $r_{1}$ is 0.03 m. (**f**) The over-segmentation result of *Monstera deliciosa* when the value of K is 100 and $r_{1}$ is 0.05 m; (**g**) the result when K is 40 and $r_{1}$ is 0.05 m; and, (**h**) the result obtained when K is 40 and $r_{1}$ is 0.03 m. (**j**) The over-segmentation result of *Calathea makoyana* when the value of K is 100 and $r_{1}$ is 0.05 m; (**k**) the result when K is 40 and $r_{1}$ is 0.05 m; and, (**l**) the result obtained when K is 40 and $r_{1}$ is 0.03 m.

Facet over-segmentation consists of two parts. The first part mainly selects seed points and carries out the coarse facet generation in the preprocessed plant point cloud. The second part conducts local K-means clustering to refine boundaries of facets. The threshold $r_{1}$ roughly defines the spacing between two different seed points in 3D space. However, coarse planar facet generation processes each point only once; namely, when the current seed point has grown into a facet, the remaining seeds can only continue to grow on the remaining points in the point cloud. Thus, the coarse facets may not have the suitable structure and points in the point cloud may not be assigned to the correct seed points. Therefore, it is crucial to refine the boundaries of the facets by applying local K-means clustering, which automatically classifies each point to its closest facet. Local K-means clustering also updates the seed points (centers) of the refined new facets, respectively. The two parts combined can generate regular and uniform facets on plant point clouds. The granularity of the generated facets can be controlled by parameters K and $r_{1}$. K is the number of points in the neighborhood when performing IPCA, which not only influences the normal of the fitted plane, but also influences the number of generated seed points (i.e., the number of facets). The smaller the value of K, the more seed points are generated. The parameter $r_{1}$ controls the minimum distance between seed points after the generation of coarse facets. Therefore, a smaller $r_{1}$ represents more facets input for local K-means clustering. Figure 5b shows the over-segmentation result of *Epipremnum aureum* when K is 100 and $r_{1}$ is 0.05 m; Figure 5c shows the over-segmentation when K is 40 and $r_{1}$ is 0.05 m; and, Figure 5d demonstrates the results when K is 20 and $r_{1}$ is 0.03 m. Figure 5f shows the over-segmentation result of the *Monstera deliciosa* point cloud when K is 100 and $r_{1}$ is 0.05 m; Figure 5g displays the result when K is 40 and $r_{1}$ is 0.05 m; and, Figure 5h is the result when K is 40 and $r_{1}$ is 0.03 m. Figure 5j shows the over-segmentation result of the *Calathea makoyana* point cloud when the value of K is 100 and $r_{1}$ is 0.05 m; Figure 5k shows the result when K is 40 and $r_{1}$ is 0.05 m; and, Figure 5l is the result obtained when K is 40 and $r_{1}$ is 0.03 m. It can be clearly seen that the granularity of the facets changes as K and $r_{1}$ decrease, and that a smaller K or $r_{1}$ tends to produce smaller facets.

### 3.3. Result of Individual Leaf Segmentation based on Facet Region Growing

**Figure 6:**
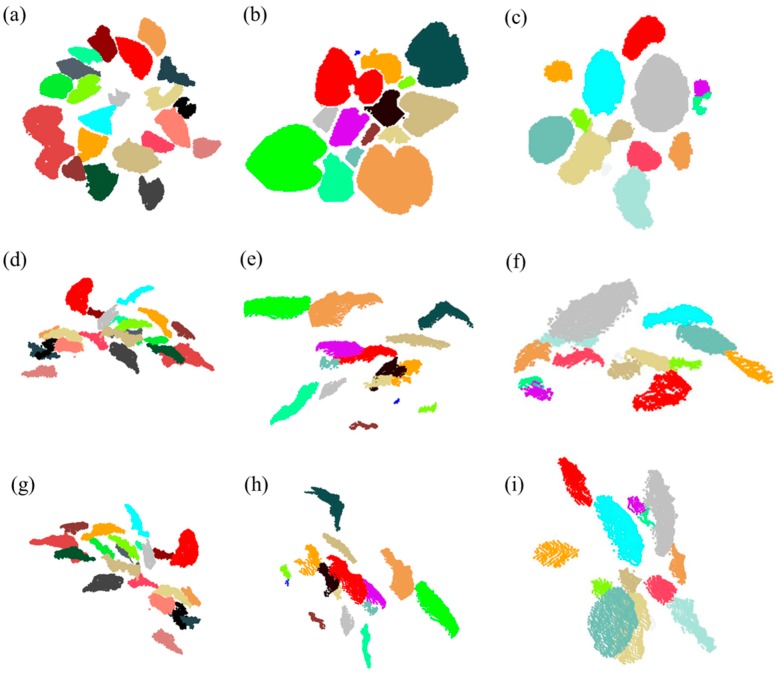
The results of individual leaf segmentation of *Epipremnum aureum, Monstera deliciosa, and Calathea makoyana*. The left column (**a**,**d**,**g**) shows three different views of the result of the point cloud of *Epipremnum aureum*, respectively. The middle column (**b**,**e**,**h**) shows three different views of the result of the point cloud of *Monstera deliciosa*, respectively. The right column (**c**,**f**,**i**) shows three different views of the result of the point cloud of *Calathea makoyana*, respectively.

The result of facet region growing is mainly influenced by the parameter $\sigma_{2}$, which is used to prevent the growing region from extending to nearby leaves. The distance from the center of an adjacent facet to the current facet needs to be less than $\sigma_{2}$ when carrying out the breadth-first-search-based facet region growing. The result of individual leaf segmentation of the point cloud of *Epipremnum aureum* is shown in three different views in Figure 6a,d,g, in which $\sigma_{2}$ is 0.0055 m. The individual leaf segmentation results of the point cloud of *Monstera deliciosa* in three different views are shown in Figure 6b,e,h, where $\sigma_{2}$ is set as 0.0025 m. The individual leaf segmentation results of the point cloud of *Calathea makoyana* in three different views are shown in Figure 6c,f,i, where $\sigma_{2}$ is set as 0.0055 m (the same as for *Epipremnum aureum*).

### 3.4. Parameters

The parameters that appear in the proposed method are listed in Table 1. The parameters can be divided into two parts. The first part contains the filtering parameters, including r, $n_{1}$, k, and $n_{2}$. The second part contains the segmentation parameters, that is, the last six parameters. The parameter values for *Epipremnum aureum*, *Monstera deliciosa, and Calathea makoyana* are different, which is due to the different leaf types of the three species. 

The *Epipremnum aureum* plant has small and curved leaves and a crowded canopy, therefore we have to apply three different filters—the z-axis filter, the number-of-points-in-a-radius filter, and the statistical average spacing filter—together to remove the outliers. So, for a plant that has small and curved leaves, we strongly recommend applying all of the four filtering parameters to the point cloud. Conversely, for a plant with large and broad leaves, such as the *Monstera deliciosa* and *Calathea makoyana*, only two filters should be applied; the z-axis filter and the number-of-points-in-a-radius (or the statistical average spacing filter) are enough to generate a satisfactory point cloud. Larger leaves usually represent a simpler canopy structure because the area of leaves is already large enough to capture energy from the sun, and a simple canopy structure means that the leaves are easier to be segmented. In addition, we have also tested the number-of-points-in-a-radius filter and the statistical average spacing filter separately on the *Monstera deliciosa* sample plant, and the two processed point clouds are nearly identical. In our experience, the values for the four filtering parameters should be set to larger values on a plant with small and dense leaves.

There are not many differences in fixing the segmentation parameters for the three different species. Only the parameter K should be decided according to the average leaf size of the plant. For a plant with small leaves (such as *Epipremnum aureum*), we use a smaller K to generate enough facets for each single leaf. Conversely, for a plant with large leaves (such as *Monstera deliciosa* and *Calathea makoyana*), we use a larger K to avoid generating too many tiny pieces of facets on a single leaf. The parameters r, $r_{1}$ and $\sigma_{2}$ are related to the precision of the scanned point cloud. The average spacing of the dense points in the sample clouds is about 1.95 mm, while the spacing of sparse points is between 3 mm and 6 mm. Thus, all the distance thresholds are tuned with a small step of 0.5 mm to avoid producing unstable fluctuations in the segmentation result.$n_{1}$, k, and K are the number thresholds that relate to the number of points in the point cloud. On average, there are about 400 to 500 points on a single leaf. Therefore, all the number thresholds are tuned with a small step of 5 to avoid producing unstable fluctuations in segmentation result.

**Figure 7:**
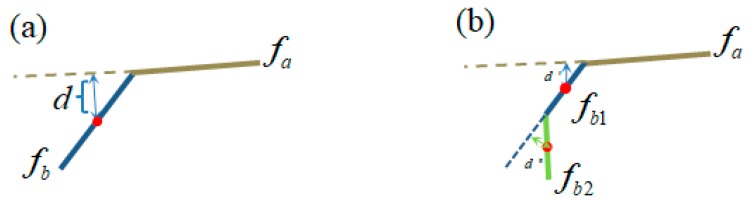
The granularity of over-segmentation affects the result of facet region growing. (**a**) shows the side view of a facet set containing only two adjacent facets, and (**b**) shows the side view of a facet set containing three adjacent facets. If the facet $f_{b}$ in (**a**) breaks into two parts, $f_{b1}$ and $f_{b2}$; In (**b**), the distance from $f_{b1}$ to $f_{a}$ is much smaller than the distance from $f_{b}$ to $f_{a}$, making the region growing to be easier.

It is also worth noting that if the facets are smaller, then the facet region growing will be easier because the distance from a facet center to another facet is smaller. This phenomenon is demonstrated in Figure 7. When $f_{b}$ breaks into two parts $f_{b1}$ and $f_{b2}$, the distance from $f_{b1}$ to $f_{a}$ is much smaller than the distance from $f_{b}$ to $f_{a}$. Therefore, if the granularity of over-segmentation is changed, the parameters for facet region growing should also be altered to avoid falsely combining facets on different leaves.

### 3.5. Performance Evaluation

Individual leaves are calculated according to the facet growing result of the proposed method. We define the following metrics to better evaluate the proposed method, and the calculated metrics are analyzed at the individual leaf level.

- TP (True Positive): if a segmented leaf region covers more than 70% of the total number of the points of the real single leaf, the segmented leaf is then regarded as a TP.
- FP (False Positive): if two real leaves are segmented by the same segmentation region, then we regard it to be an FP.
- FN (False Negative): If more than 70% points of a real leaf are not covered by any segmentation, then we call it an FN.

Because we only provide point clouds of leaves for evaluating the proposed method, the True Negative (TN) does not exist. Based on the above definitions of TP, FP, and FN, we calculate three metrics—namely, the recall, precision, and F-measure—to quantitatively evaluate the proposed algorithm. The three metrics are explained as follows:

(3) $$\mathrm{recall}=\frac{\mathrm{TP}}{\mathrm{TP}+\mathrm{FN}}\times 100\%$$

(4) $$\mathrm{precision}=\frac{\mathrm{TP}}{\mathrm{TP}+\mathrm{FP}}\times 100\%$$

(5) $$F-\mathrm{Measure}=\frac{2\mathrm{TP}}{2\mathrm{TP}+\mathrm{FP}+\mathrm{FN}}\times 100\%$$

**Figure 8:**
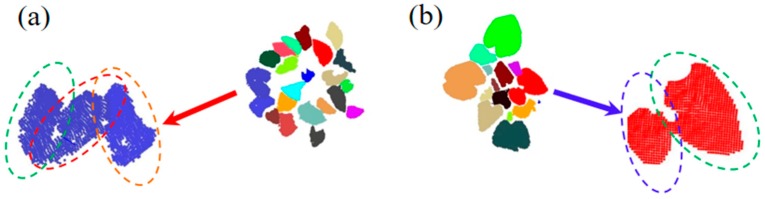
False Positives (FP) in two sample point clouds: (**a**) the two FPs for the point cloud of *Epipremnum aureum*; (**b**) the FP of the point cloud of *Monstera deliciosa* sample plant.

Table 2 lists the evaluation results of the proposed method on the three sample point clouds by these defined metrics. The proposed method successfully segmented 21 individual leaves from the point cloud of *Epipremnum aureum* with 23 real leaves, and segmented 15 individual leaves from the point cloud of *Monstera deliciosa* with 16 real leaves. The proposed method also correctly segmented 11 individual leaves from the point cloud of *Calathea makoyana* with 12 real leaves. The average precision of the method is higher than 90%, and the F-measure is higher than 95% on all three types of plants. The metrics show that the proposed method is able to obtain high TP numbers in the three cases. The FPs are mainly attributed to spatially adjacent leaves. For example, several leaves are overlapping and coplanar, making them extremely difficult to be correctly segmented even by a human (as illustrated in Figure 8a,b). We also manually evaluated the cover rates (defined as the ratio of a segmented leaf area to the original leaf area) of the three sample plants. The average cover rate for the *Epipremnum aureum* sample plant is 94.35%; the maximum cover rate among all leaves is 100%, and the minimum is about 70.00%. The average cover rate of *Monstera deliciosa* roughly reaches 100% because our method generates almost perfect segmentation result on plants with large leaves. The average cover rate of *Calathea makoyana* is 92.08%; the maximum cover rate among all leaves of *Calathea makoyana* is 100%, and the minimum cover rate is about 50%.

The proposed method took 13.04 s to complete the individual leaf segmentation on the point cloud of *Epipremnum aureum,* 4.85 s on the point cloud of *Monstera deliciosa,* and 4.50 s on the point cloud of *Calathea makoyana*. Thus, the proposed method can be used for quasi-real-time plant phenotyping.

**Figure 9:**
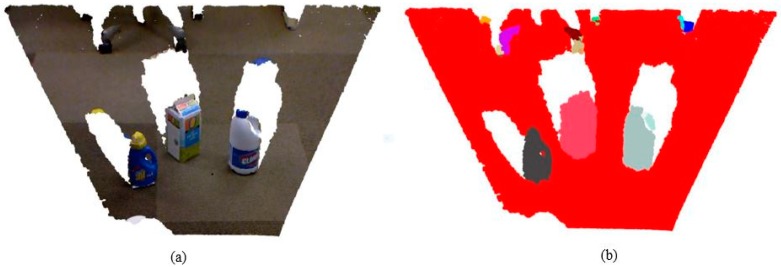
The segmentation result of our method on a point cloud containing a table surface and several objects (bottles and boxes): (**a**) the original point cloud with real colors, and (**b**) the segmentation result with different objects labeled in different colors, respectively.

Although the original goal of the proposed method was to segment individual leaves from a point cloud, we found that it has the potential to be applied to many applications that aim to segment regular surfaces and objects from a point cloud; for example, remote sensing, building information modeling (BIM), and simultaneous localization and mapping. In Figure 9, we apply the segmentation method to a point cloud containing a table surface and several objects (bottles and boxes), and obtain a satisfactory surface segmentation result, where all standing objects are correctly segmented from the table surface.

## 4. Conclusions

In order to address the problems of image-based leaf segmentation methods, such as restrictions on species and vulnerability toward overlapping leaves, we propose a new individual leaf segmentation method for plant point clouds. The method can be divided into three steps: pre-processing, facet over-segmentation, and facet region growing. Our method is not only suitable for the point clouds generated from two-camera stereo systems, but also handles the plant point clouds scanned from structured light sensors. In a performance assessment, our method achieved higher than 90% in average recall and higher than 95% in average F-measure for three different kinds of greenhouse ornamental plants. Although the original goal of our method was to segment individual leaves from a point cloud, we found that it has the potential to be applied to many applications that aim to segment regular surfaces and objects from a point cloud; for example, remote sensing, building information modeling (BIM), and simultaneous localization and mapping.

In this research, we only handled the plant point clouds that are sufficiently dense, which requires that a leaf should contain at least 50 points. Leaves that are both coplanar and adjoining are extremely difficult to segment, even for a human. For our approach, the FPs (False Positives) which stem from incorrectly growing facets across adjacent leaves are unavoidable. Although a FP segmentation does not influence the total leaf area computed, it reduces the number of detected leaves in the point cloud, resulting in an increase in the average leaf area index. For future research, we are interested in integrating popular deep learning algorithms into the 3D leaf segmentation method to separate each individual leaf from heavily clustered foliage. We also plan to test the proposed method on the plant point clouds scanned by many other kinds of 3D sensors with distinct accuracies.

## Figures and Tables

**Table 1:** Parameter settings for the three different point clouds.

Parameter	Description	Value for *Epipremnum*	Value for *Monstera*	Value for *Makoyana*
r	The radius parameter of the search sphere used for removing outliers.	0.015 m	—	0.015 m
$n_{1}$	A threshold that defines the minimum number of points in the search sphere.	85	—	50
k	The number of the nearest neighbors of the current point when computing the average spacing.	40	25	—
$n_{2}$	A threshold that is used to multiply the standard deviation of the average spacing.	1	0.1	—
K	The number of the nearest neighbor points used in IPCA.	20	40	40
$r_{1}$	A radius threshold used for coarse planar facet generation.	0.03 m	0.03 m	0.03 m
θ	An angle threshold for comparing two normals.	$23^{\circ }$	$23^{\circ }$	$23^{\circ }$
$\sigma_{1}$	A threshold for measure the distance from a point to a plane.	0.025 m	0.025 m	0.025 m
$r_{2}$	A radius threshold used in local K-means clustering.	0.1 m	0.1 m	0.1 m
$\sigma_{2}$	A threshold that defines the distance from the center of one facet to another adjoining facet.	0.0055 m	0.0025 m	0.0055 m

**Table 2:** Quantitative results of the proposed method.

Plant Type	True Positive (TP)	Reference	False Positive (FP)	False Negative (FN)	Recall	Precision	F-Measure
*Epipremnum aureum*	21	23	2	0	100%	91.30%	95.45%
*Monstera deliciosa*	15	16	1	0	100%	93.75%	96.77%
*Calathea makoyana*	11	12	0	1	91.67%	100%	95.65%
